# The comparative impact of central vs. peripheral VA-ECMO cannulation on postoperative graft dysfunction in lung transplantation: a retrospective analysis

**DOI:** 10.3389/fcvm.2025.1512742

**Published:** 2025-02-25

**Authors:** Xiaowen Wu, Shuai Miao, Yan Zhou, Tianjun Wu, Jingyu Chen, Guilong Wang, Xin Zhang

**Affiliations:** ^1^ Department of Anesthesiology, The Affiliated Wuxi People’s Hospital of Nanjing Medical University, Wuxi People’s Hospital, Wuxi Medical Center, Nanjing Medical University, Wuxi, Jiangsu, China; ^2^ Department of Thoracic Surgery, The Affiliated Wuxi People’s Hospital of Nanjing Medical University, Wuxi People’s Hospital, Wuxi Medical Center, Nanjing Medical University, Wuxi, Jiangsu, China; ^3^ Department of Anesthesiology, Duke University School of Medicine, Durham, NC, United States

**Keywords:** lung transplantation, anesthesia, retrospective study, extracorporeal membrane oxygenation, primary graft dysfunction, survival rate

## Abstract

**Background:**

Lung transplantation (LTx) is the definitive treatment for end-stage pulmonary diseases, with venoarterial extracorporeal membrane oxygenation (VA-ECMO) used as a common perioperative support. However, it remains unclear if central (cVA-ECMO) or peripheral (pVA-ECMO) cannulation routes yield better outcomes in postoperative prognosis. This study compares the impact of these two cannulation strategies on primary graft dysfunction (PGD) incidence in LTx patients.

**Methods:**

A retrospective analysis was performed on 153 LTx patients supported with VA-ECMO at the Wuxi Lung Transplant Center (January 2019–March 2023). Patients were divided into central (*n* = 31) and peripheral (*n* = 91) groups. Data included recipient/donor demographics, preoperative status, and follow-up outcomes. The primary outcome was PGD within 72 h after reperfusion, whereas secondary outcomes included in-hospital mortality, 1-year survival, renal support needs, ventilation duration, intensive care unit (ICU) stay, and biochemical markers.

**Results:**

PGD incidence was significantly higher in the peripheral group, with longer ECMO duration, ventilation, and ICU stay. Central VA-ECMO showed advantages in in-hospital mortality and 1-year survival rates.

**Conclusion:**

Central VA-ECMO cannulation may reduce postoperative complications and improve survival for LTx recipients. Prospective studies are needed to confirm these findings and refine perioperative ECMO management.

## Introduction

Lung transplantation (LTx) represents the definitive treatment for end-stage pulmonary diseases, including chronic obstructive pulmonary disease (COPD) and pulmonary hypertension from various etiologies (1–3). For patients unable to tolerate one-lung ventilation or those who experience hemodynamic disturbances during LTx, cardiopulmonary bypass is essential for perioperative support (1, 4). However, since 2001, advancements have increased the use of extracorporeal membrane oxygenation (ECMO), while most LTx centers have transitioned from CPB to ECMO for mechanical life support since 2008 (5–11). Among ECMO types, venoarterial ECMO (VA-ECMO) showed effective respiratory and circulatory support during LTx.

VA-ECMO supports patients with moderate-to-severe pulmonary hypertension perioperatively and is classified into peripheral and central types based on cannulation location. Peripheral VA-ECMO is less invasive, but it may limit oxygen delivery and hemodynamic stability due to reliance on the patient's lung function. In contrast, central VA-ECMO offers direct, fully oxygenated blood flow, providing better oxygenation and stable circulation, which makes it more suitable for high-risk lung transplant cases despite being more invasive. However, the current lack of high-quality randomized controlled trials and clinical data highlights the potential for selection bias and uncertainty about differences in perioperative management and prognosis between these approaches.

Primary graft dysfunction (PGD) manifests as a spectrum of mild-to-severe lung injury occurring within the first 72 h after LTx, representing a major cause of early morbidity and mortality. PGD is characterized by progressive hypoxemia and alveolar infiltrates on a chest radiography (12). Although no multicenter study revealed a significant correlation between PGD incidence and transplantation surgery (13), some studies still suggested that ECMO support may mitigate the risk of severe PGD and enhance patient survival rates (1, 5).

Therefore, we planned a retrospective analysis of existing data from our center to compare the impact of central (right atrium–ascending aorta) vs. peripheral (femoral artery) venous VA-ECMO cannulation on PGD outcomes.

## Methods and materials

### Patients

**Figure 1:**
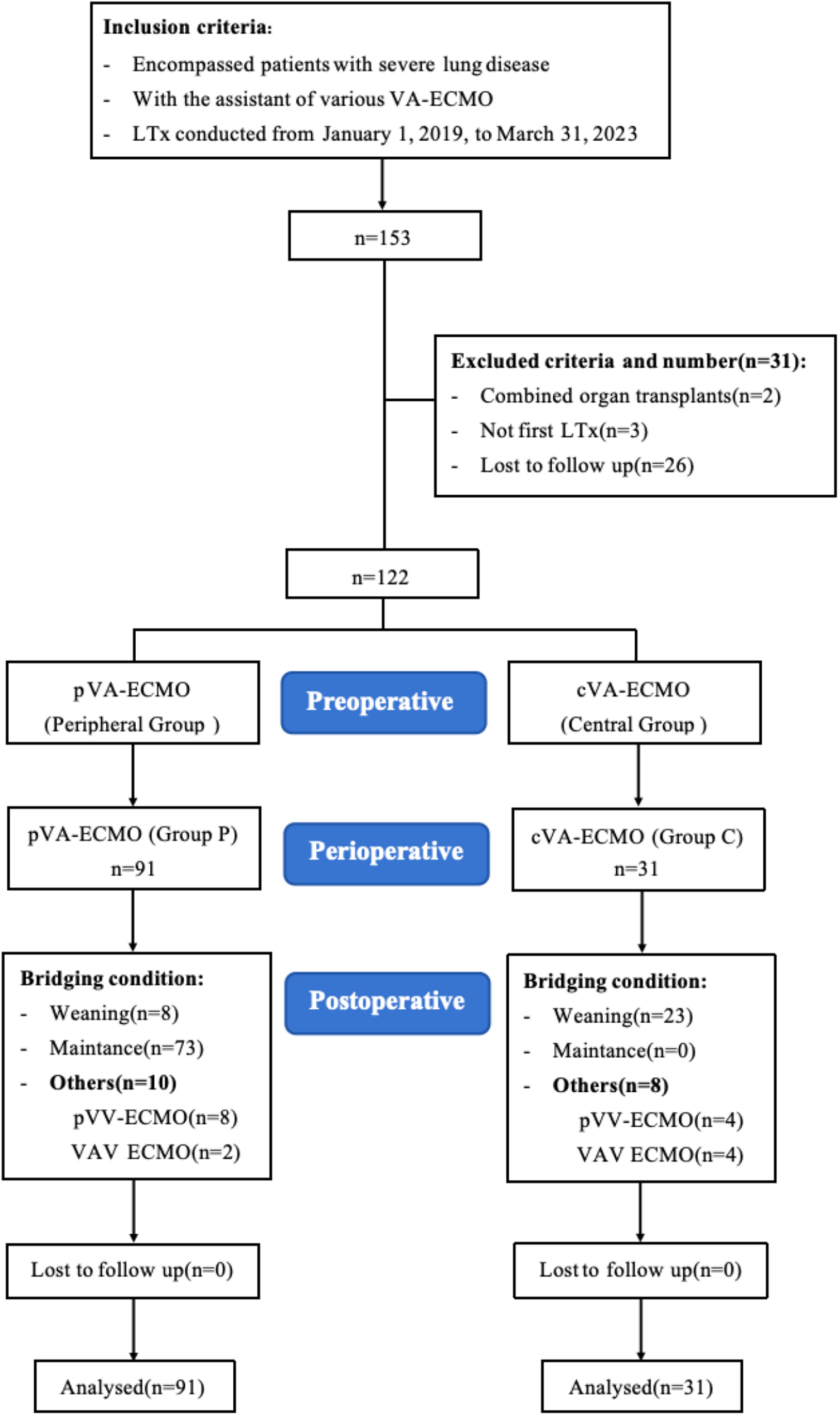
The flowchart of inclusion and exclusion criteria and grouping of participants.

The Ethics Review Committee of Wuxi People's Hospital approved this study (IRB number: KY21064), which included 153 patients with severe lung disease who underwent LTx with VA-ECMO assistance at the Wuxi Lung Transplant Center from January 1, 2019, to March 31, 2023. Cases with combined organ transplantation, non-first-time LTx, bridging ECMO support before surgery, or loss to follow-up were excluded (Figure 1). Informed consent was waived due to the retrospective nature of the study. Organ donation and transplantation followed the regulations of the Jiangsu Organ Transplantation Committee and the Declaration of Helsinki, with informed consent obtained from donors or their authorized representatives. All cadaveric donors at the Wuxi Organ Transplantation Center have been brain-dead patients since 2015.

Data on recipient variables [gender, age, body mass index BMI), diagnosis, and mean pulmonary artery pressure (MPAP)], donor variables (gender, age, BMI, donation type, oxygenation index, and ventilation days), and operative characteristics [operation duration, cold ischemia time (CIT), mean arterial pressure < map > during surgery and 3 days postoperatively, fluid balance (intake volume−output volume), vasoactive drug dosages, and oxygenation index] were collected. PGD is a clinical syndrome of acute lung injury occurring within the first 72 h after LTx, characterized by hypoxemia and alveolar infiltrates on chest x-ray (CXR) (14, 15). PGD is graded based on the oxygenation index [the PaO_2_/FiO_2_ (P/F) ratio] and radiographic findings using a scale from 0 (no disease) to 3 (severe PGD). CIT was defined as the duration from the initiation of *ex vivo* perfusion of the donor lung to the opening of the pulmonary artery in the transplanted lung, with specific distinctions between single and double lung transplants (16).

The primary outcome measured was the occurrence of PGD grade 3 within 72 h. Post-reperfusion PGD grade was assessed according to the ISHLT2016: PGD is classified as grade 3 when PaO_2_/FiO_2_ is <200 with definite radiographic infiltrates of pulmonary edema. Additionally, using extracorporeal life support (ECLS) postoperatively was considered as a possible factor (12, 17).

Secondary outcomes comprised early indicators, such as the need for continuous renal replacement therapy (CRRT), postoperative ventilation duration, and length of stay in the intensive care unit (ICU). In-hospital mortality and 1-year postoperative survival rates were also assessed. Additionally, biomarkers including N-terminal pro-b-type natriuretic peptide (NT-proBNP), troponin T, and albumin levels 72 h after surgery were measured.

Indications for establishing VA-ECMO included (1) hypoxemia; (2) high pulmonary arterial pressure (>50% of systemic blood pressure); (3) right ventricular or biventricular failure; (4) other conditions such as acute chronic heart failure, hypothermia, and cardiac arrest with ongoing cardiopulmonary resuscitation (ECPR) (18–20). The choice between peripheral or central cannulation depended on the surgeon's preference.

The study cohort was divided into two groups based on the ECMO cannulation strategy: (1) the central cannulation group (cVA-ECMO), with inflow through the ascending aorta, and (2) the peripheral group (pVA-ECMO), with inflow through the femoral artery.

### Anesthesia management and cannulation

All patients underwent radial artery catheterization for blood pressure monitoring before anesthesia induction. General anesthesia was induced using titrated midazolam (0.1 mg/kg), sufentanil (0.5–1 μg/kg), propofol (0.5–1 mg/kg), and rocuronium (0.5–1 mg/kg). A double-lumen endotracheal tube, sized according to the patient's height, was used for intubation. Lung protective ventilation strategies were applied to recipients, utilizing a low tidal volume of ≤6 ml/kg predicted body weight (PBW) for mechanical ventilation, adjusted based on donor characteristics. Intraoperatively, we maintained the minimum FiO₂ necessary to achieve an appropriate PaO₂ > 70 mmHg and hemoglobin oxygen saturation (SpO₂) ≥ 92%, while ensuring PaCO₂ remained within the pre-transplantation range. A Swan-Ganz catheter was placed via the right internal jugular vein after the induction to measure PAP, and cardiac function was assessed using transesophageal echocardiography (TEE). Anesthesia was maintained using propofol, sufentanil, and rocuronium, with vasoactive drugs administered as needed to maintain hemodynamic stability.

The ECMO team, including the anesthesiologist and surgeon, determined ECMO use based on the patient's history and anesthesia assessment. cVA-ECMO involved thoracotomy and cannulation of the right atrium and ascending aorta, while pVA-ECMO was established via the femoral artery and vein cannulation using the Seldinger technique. ECMO was initiated to maintain central venous pressure of >5 mmHg without a significant decrease.

Arterial blood gas and activated clotting time (ACT) were monitored every 30 min, with heparin administered to maintain ACT between 180 and 200 s. TEE was used to assess volume status, and surgery proceeded with ventilation using small tidal volumes (5–6 ml/kg) and low airway pressures (15–25 cm H_2_O).

After surgery, fluid management and ECMO weaning were based on circulation and oxygenation assessments. VA-ECMO was removed when stable hemodynamics was achieved, the oxygenation index exceeded 300, and echocardiographic evaluations showed adequate left ventricular contractility (aortic velocity-time integral >10 and left ventricular ejection fraction (LVEF) > 30%]. Otherwise, VA-ECMO was maintained, with pVA-ECMO continuing in the ICU or converting cVA-ECMO to pVA-ECMO if needed.

### Statistical analysis

Statistical analysis was conducted using SPSS 25.0 and Stata software to assess the relationship between cannulation strategy and PGD. Demographic and perioperative factors were analyzed. Continuous variables that followed a normal distribution were presented as a mean ± standard deviation (SD). Continuous variables not conforming to a normal distribution were expressed as a median and interquartile range (IQR), whereas categorical variables were presented as percentages (%). The *t*-test or Mann–Whitney *U*-test was used for continuous variables, and the chi-square or Fisher's exact test for categorical variables.

A multivariable logistic regression model was applied to adjust for inclusion criteria and assess in-hospital mortality. One-year postoperative mortality was analyzed using Cox regression. Subgroup analysis was used to evaluate survival in the central cannulation group. Incidence rates and Kaplan–Meier survival curves were utilized to compare differences between the two cannulation strategies. A *p* < 0.05 indicated statistical significance.

## Results

A total of 495 patients underwent LTx at our institution from January 2019 to March 2023. We included 122 patients who used VA-ECMO during surgery, excluding those with combined organ transplants, non-first LTx, or patients lost to follow-up. Among these, 91 (74.6%) patients underwent pVA-ECMO, while 31 (25.4%) patients underwent cVA-ECMO.

Table 1 shows patients' demographic characteristics and donor data. No significant differences regarding gender, age, donation types, or general condition were observed between the two groups. The average age of the central group was 57 years, which was slightly older than that of the peripheral group (54 years; *P* = 0.871). Both groups had normal BMI values, with the central group showing a slightly lower BMI (19.69 vs. 20.94 kg/m^2^, *P* = 0.216). Clinical diagnosis demonstrated no statistical difference (*P* = 0.221), with interstitial pulmonary fibrosis (IPF) being the most common condition.

**Table 1:** Patients’ demographic characteristics of patients and donor data.

Variable	*P* (*n* = 91)	C (*n* = 31)	*p*-value
Female sex (%)	25 (27.5)	9 (29.0)	0.867
Age (years)	54 (41–63)	57 (35–62)	0.871
Weight (kg)	58.44 ± 12.504	52.74 ± 17.115	0.096
Height (cm)	168 (160–171)	168 (162–172)	0.984
BMI (kg/m^2^)	20.94 ± 3.88	19.69 ± 3.44	0.216
Diagnosis			0.221
IPF	41 (45.1)	18 (58.1)	
Pneumoconiosis	18 (19.8)	2 (6.5)	
COPD	10 (11.0)	6 (19.4)	
Bronchiectasis	10 (11.0)	2 (6.5)	
Other	12 (13.2)	3 (9.7)	
Donor Data			
Sex, *n* (%)			0.242
Male	57 (62.6)	23 (74.2)	
Female	34 (37.4)	8 (25.8)	
Age (year)	38.0 ± 11.8	37.2 ± 12.1	0.566
BMI (kg/m^2^)	23.2 ± 2.9	23.4 ± 3.1	0.975
Types of Donation, *n* (%)			0.895
DBD	82 (90.1)	27 (87.1)	
DCD/DBCD	9 (9.9)	4 (12.9)	
Oxygenation index	428.8 ± 89.5	430.0 ± 88.1	0.353
Ventilation (days)	4.0 (3–7)	3.8(3–6.5)	0.478

Values are expressed as mean ± SD, median (IQR), or *n* (%).

SD, standard deviation; IQR, interquartile range; BMI, body mass index; IPF, interstitial pulmonary fibrosis; COPD, chronic obstructive pulmonary disease.

Table 2 summarizes patients’ clinical characteristics. In the pVA-ECMO group, 65 patients (74.6%) underwent double LTx compared to 28 (90.3%) patients in the central group. Surgeries were performed by three surgeon groups with significant differences in central cannulation rates (*P* = 0.006). Regarding the incision type, 84 patients underwent thoracotomy, with 79 in the peripheral group and 5 in the central group. The remaining 38 cases were performed using the clamshell approach with a significant difference in distribution (*P* < 0.0001).

**Table 2:** Summary of the patients’ clinical characteristics.

Variable	*P* (*n* = 91)	C (*n* = 31)	*p*-value
LTx type			0.033
Single LTx	26 (28.6)	3 (9.7)	
Double LTx	65 (71.4)	28 (90.3)	
Surgeon group			0.006
Group A	22 (24.2)	17 (54.8)	
Group B	34 (37.4)	6 (19.4)	
Group C	35 (38.5)	8 (25.8)	
Surgical incision			< 0.0,001
Sternum sparing	79 (86.8)	5 (16.1)	
Clamshell	12 (13.2)	26 (83.9)	
Ischemic time (min)			
Double LTx			
First lung	411.98 ± 77.92	389 ± 100.64	0.236
Second lung	564.45 ± 92.82	498.39 ± 109.34	0.004
Single LTx	414 ± 90.58	450 ± 51.96	0.509
Pulmonary artery pressure (mmHg)			
Before ECMO establishment			
SPAP	71.29 ± 23.10)	75.58 ± 26.18	0.389
MPAP	45 (36–59.25)	49 (38–58)	0.351
ECMO bypass initiation			
SPAP	49.06 ± 19.89	34.71 ± 13.68	<0.001
MPAP	35.11 ± 15.81	25.26 ± 9.18	<0.001
Intraoperative ECMO volume (L/min)	2.17 (2–2.5)	3.5 (3–4)	<0.001
PaO_2_/FiO_2_ (T0)	267.97 ± 133.29	426.67 (254–521.67)	<0.001
PGD (T0)	54 (59.3)	8 (25.8)	<0.001
Grade 1–2	45 (49.5)	8 (25.8)	0.477
Grade 3	9 (9.8)	0 (0)	
Postoperative ECMO			
Bridging			<0.0001
Weaning off	8 (8.8)	23 (74.2)	
pVA-ECMO	73 (80.2)	4 (12.9)	
pVV-ECMO	8 (8.8)	4 (12.9)	
VAV-ECMO	2 (2.2)	0 (0)	
Postoperative ECMO duration (days)	2 (1–4)	0.65 ± 1.49	< 0.0001
Operation duration (min)	378.98 ± 117.46	378.58 ± 107.09	0.987
Fluid balance (ml)	246.92 ± 940.34	641.77 ± 1,379.99	0.078
Albumin infusion (g)	150 (60–250)	269.19 ± 150.19	< 0.001
Urine volume (ml)	2,200 (1,500–3,000)	2,000(1,400–2,500)	0.087

Values are expressed as mean ± SD, median (IQR), or *n* (%).

SD, standard deviation; IQR, interquartile range; SPAP, systolic pulmonary artery pressure; MPAP, mean pulmonary artery pressure.

CIT was recorded for each transplanted lung in bilateral cases. No significant difference was found for the first transplanted lung (*P* = 0.236). However, the CIT for the second lung was significantly longer in the peripheral group (564.45 vs. 498.39 min, *P* = 0.004), possibly due to different surgical incisions. For single lung transplantation, CITs were similar between the groups (*P* = 0.509).

PAP was monitored continuously during surgery using a Swan-Ganz catheter. Systolic PAP (SPAP) and MPAP were recorded before and after initiating ECMO. Before ECMO initiation, SPAP and MPAP were similar between the groups, showing no significant differences. After ECMO initiation, both SPAP and MPAP significantly decreased in both groups, with a greater reduction in the central group (SPAP: pVA-ECMO 49.06 vs. cVA-ECMO 34.71 mmHg, *P* < 0.001; MPAP: pVA-ECMO 35.11 vs. cVA-ECMO 25.26 mmHg, *P* < 0.001). The central group also had a significantly higher perioperative diversion flow rate (pVA-ECMO 2.17 L/min vs. cVA-ECMO 3.50 L/min, *P* < 0.001).

Upon the time after the operation (T0), PGD incidence in the peripheral group was significantly higher than that in the central group [pVA-ECMO 54 (59.3%) vs. cVA_ECMO 8 (25.8%), *P* < 0.001]. Among these patients, there were 9 cases of severe PGD in the peripheral group and 0 cases in the central group (*P* = 0.477).

The need for postsurgical VA-ECMO support was also assessed. In the peripheral group, 8 patients (8.8%) were weaned off ECMO in the operating room, 73 (80.2%) patients retained pVA-ECMO, 8 (8.8%) patients were switched to VV-ECMO, and 2 patients were switched to VAV-ECMO before transfer to an ICU. In contrast, 23 patients (74.2%) were weaned off ECMO in the operating room, 4 (12.9%) patients were switched to pVA-ECMO, and another 4 (12.9%) patients were switched to VV-ECMO before transfer to an ICU in the central group. The difference in ECMO weaning and support needs between the groups was statistically significant (*P* < 0.0001).

The postoperative ECMO duration was significantly longer in the peripheral group compared to the central group [pVA-ECMO: 2 (1, 4) days vs. cVA-ECMO: 0.65 (1.49) days, *P* < 0.0001]. Operation duration, fluid balance, and urine output showed no significant differences between the groups. However, the central group required more albumin infusion (pVA-ECMO: 150 g vs. cVA-ECMO: 269.19 g, *P* < 0.001).

The incidence and severity of PGD in the ICU were assessed at multiple time points after LTx (Table 3). On day 0, grade 3 PGD was seen in 31 patients (34.1%) in the peripheral group and 8 patients (34.8%) in the central group (*P* = 0.059). On day 1, 23 patients (25.3%) in the peripheral group and 2 patients (6.5%) in the central group had grade 3 PGD (*P* = 0.0025). On day 2, grade 3 PGD was observed in 24 patients (26.4%) in the peripheral group and 1 patient (3.2%) in the central group (*P* = 0.006). Over 72 h, the peripheral group had 57 cases of grade 3 PGD compared to 8 cases in the central group (*P* < 0.0001).

**Table 3:** Patients’ follow-up and survival data.

Variable	P (*n* = 91)	C (*n* = 31)	*p*-value
Postoperative PGD grade 3			
Day 0	31 (34.1)	5 (16.1)	0.059
Day 1	23 (25.3)	2 (6.5)	0.025
Day 2	24 (26.4)	1 (3.2)	0.006
Day 3	21 (23.1)	4 (12.9)	0.226
72 h total PGD grade 3(%)	57 (62.6)	8 (25.8)	5.05*10 ^−13^
Postoperative PaO_2_/FiO_2_			
Day 0	257.5 (176–353)	265.85 ± 106.74	0.981
Day 1	314.79 ± 138.34	308.29 ± 114.12	0.814
Day 2	302.99 ± 130.99	303.96 ± 102.99	0.97
Day 3	276.14 ± 129.06	310.8 ± 94.52	0.115
Postoperative MAP within 72 h (mmHg)			
Day 0	85.81 ± 17.39	82.57 ± 11.99	0.255
Day 1	83.59 ± 10.42	80.95 ± 12.91	0.255
Day 2	83.49 ± 7.17	81.35 ± 10.86	0.216
Day 3	84 (80–88.5)	85.97 ± 11.81	0.384
Infection	41 (45.1)	5 (16.1)	0.004
Lower limb ischemia	27 (29.7)	2 (6.5)	0.009
Early outcomes			
Ventilation time (days)	4 (2–9)	2 (1–4)	<0.001
ICU stay (days)	8 (5–13)	4 (2–7)	<0.0001
CRRT	22 (24.2)	6 (19.4)	0.581
72 h Post-surgery biomarkers			
Troponin T(ng/ml)	3.73 (2.15–5.8)	2.66 (2.05–3.27)	0.007
Albumin (g/ml)	38.83 ± 4.76	38.03 ± 3.63	0.393
NT-proBNP (pg/ml)	733.57 (1,835.63)	380.67 (247.27–2,045.5)	0.017
Short-/long-term outcome			
Thirty-day survival rate (%)	68 (74.7)	27 (87.1)	0.152
One-year survival rate (%)	60 (65.9)	24(77.4)	0.233

Values are expressed as mean ± SD, median (IQR), or *n* (%).

SD, standard deviation; IQR, interquartile range; PGD, primary graft dysfunction; NT-proBNP, brain natriuretic peptide.

The oxygenation index and MAP were comparable across all time points (days 0–3). However, the infection rate, including catheter-related and pulmonary infections, was significantly higher in the peripheral group [pVA-ECMO: 41 cases (45.1%) vs. cVA-ECMO: 5 cases (16.1%), *P* < 0.001]. Additionally, lower limb ischemia occurred in 27 patients in the peripheral group compared to 2 patients in the central group [pVA-ECMO: 27 cases (29.7%) vs. cVA-ECMO: 2 cases (6.5%), *P* < 0.01].

Postoperative ventilation and ICU stay duration were significantly longer in the peripheral group compared to the central group. The peripheral group had an average ventilation time of 4 days (range: 2–9 days) and an ICU stay of 8 days (range: 5–13 days), while the central group had an average ventilation time of 2 days (range: 1–4 days) and an ICU stay of 4 days (range: 2–7 days; ventilation time: *P* < 0.001; ICU stay: *P* < 0.0001). CRRT support demonstrated no significant differences between the groups. Regarding biomarkers, troponin T and NT-proBNP levels were higher 72 h after surgery in the peripheral group (troponin T: 3.73 ng/ml vs. 2.66 ng/ml, *p* = 0.007; NT-proBNP: 733.57 pg/ml vs. 380.67 pg/ml, *P* = 0.017). Albumin levels were similar between the groups (*P* = 0.393).

**Figure 2:**
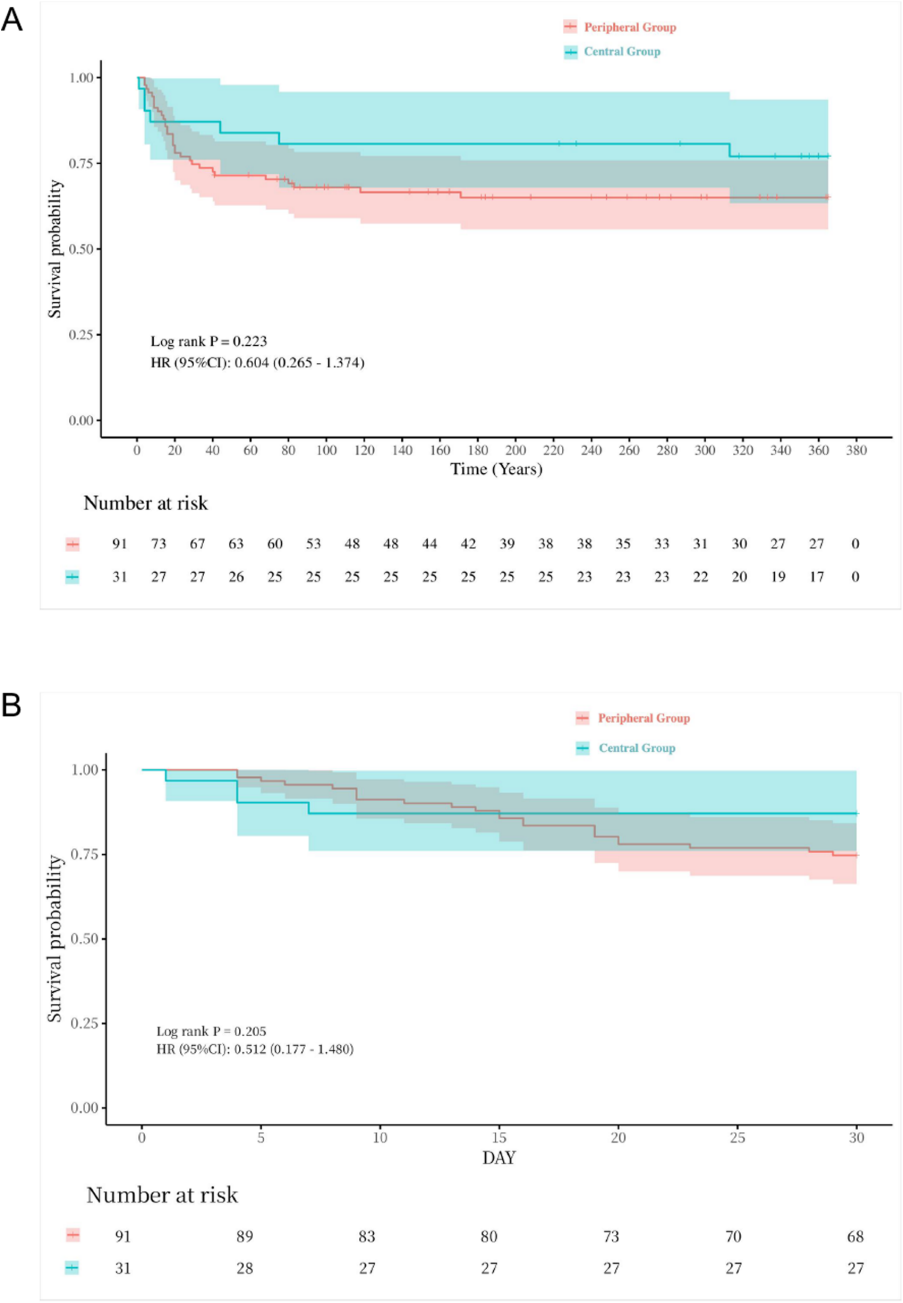
Kaplan–Meier plot for LTx one-year & thirty-day survival for all cases. **(A)** one-year survival curve, *p* = 0.233. **(B)** thirty-day survival curve, *p* = 0.152.

The 30-day survival rate was comparable between the groups (peripheral: 74.7% vs. central: 87.1%, *P* = 0.152), as was the 1-year survival rate (peripheral: 65.9% vs. central: 77.4%, *P* = 0.233). The Kaplan–Meier survival curve showed no statistically significant difference in 30-day and 1-year survival rates between the groups (Figure 2).

In the Cox regression model, the hazard of PGD grade 3 was significantly lower in the central group compared to the peripheral group (OR = 0.207, *P* = 0.001). The risk ratio further decreased to 0.179 (*P* = 0.001) after adjusting for age, gender, BMI, and bilateral lung transplantation. However, no statistically significant differences in survival rates were observed after adjusting for the surgeon team (Table 4). Regardless of adjustments, the odds ratios for 30-day and 1-year survival rates remained statistically insignificant (Table 4). Additionally, no significant differences in 30-day or 1-year survival rates were found between patients with and without PGD after adjusting for Models 1–3 (Supplementary Table S1).

**Table 4:** Thirty-day and 1-year survival rate and PGD grade 3 after adjustment .

Categories	Model 1			Model 2			Model 3		
	*P*-value	OR/HR	95% CI	*P*-value	OR/HR	95% CI	*P*-value	OR/HR	95% CI
Thirty-day survival rate									
P	Reference			Reference			Reference		
C	0.160	0.438	0.138–1.385	0.138	0.400	0.119–1.343	0.059	0.306	0.090–1.045
One-year survival rate									
P	Reference			Reference			Reference		
C	0.237	0.565	0.219–1.455	0.145	0.466	0.167–1.302	0.095	0.418	0.150–1.163
PGD Grade3									
P	Reference			Reference			Reference		
C	0.001	0.207	0.084–0.515	0.001	0.179	0.068–0.472	0.995	<0.001	

Model 1: Non-adjusted; Model 2: Adjusted for Sex, Age, BMI, Bilateral LTx; Model 3: Adjusted for Surgeon Group.

P: Peripheral Group.

C: Central Group.

## Discussion

ECMO is commonly used during cardiothoracic surgeries such as LTx, especially for patients with severe right ventricular dysfunction or hypoxia. PGD contributes significantly to short- and long-term mortality after LTx (21). This study assessed how different VA-ECMO cannulation strategies (central vs. peripheral) influence LTx outcomes, focusing on PGD as the primary measure, with 30-day and 1-year mortality as secondary outcomes. Most patients had severe pulmonary hypertension and were on VA-ECMO, making their condition more critical compared to those on other ECMO types or no extracorporeal support. This caused a more pronounced difference in their prognosis and survival rates.

The choice of VA-ECMO cannulation approach significantly impacted the primary outcome of this study. VA-ECMO stabilizes hemodynamics by bypassing the pulmonary circulation and delivering controlled blood flow to the graft during reperfusion (22). Peripheral VA-ECMO blends blood flow from the patient's lungs with ECMO, with the balance influenced by various factors. In contrast, central cannulation allows direct mixing of fully oxygenated blood with cardiac blood flow, offering stronger respiratory and hemodynamic support without relying on residual cardiac function. Our study found a lower PGD incidence in the central cannulation group compared to the peripheral group. Adjustments for variables revealed differences in PGD severity at different times, indicating that the cannulation method chosen impacts PGD development. Furthermore, the choice of cannulation type during surgery is primarily influenced by the surgeon's preference and the patient's clinical condition, while the postoperative selection is determined by factors such as oxygenation and hemodynamic stability.

A retrospective study identified elevated PAP as an independent risk factor for PGD in LTx patients with IPF, with a 64% increase in risk for every 10 mm Hg rise in PAP. Our study found that cVA-ECMO (with an average transfer flow rate of 3.5 L/min) effectively lowered PAP during pulmonary artery occlusion and reduced lung volume loading during reperfusion compared to pECMO. This reduction mitigated macrophage activation and the inflammatory cascade (23–25), contributing to a lower incidence of severe PGD in the central group, as evidenced by a higher oxygenation index before weaning.

NT-ProBNP is a well-established biomarker of left ventricular overload, commonly used to assess the degree of heart failure (26–28). It presents a significant correlation with pulmonary hypertension and cardiac insufficiency both before and after ECMO utilization according to numerous studies (29, 30). Central cannulation enhances cardiac function more effectively than peripheral ECMO, reducing pulmonary vein return and left atrial congestion. Although the left atrial pressure was not monitored, the central group showed significantly lower postoperative troponin T and NT-ProBNP levels, suggesting that cVA-ECMO may reduce myocardial ischemia-reperfusion injury and protect cardiac function.

The ischemia-reperfusion duration is a critical factor influencing the development of PGD (31). This length of ischemia is influenced by various factors, including incision size, surgeon expertise, and the use of life support technologies. Our institution uses cVA-ECMO, which resulted in shorter CIT for the second lung in bilateral LTx since it avoids the need to close one side before opening the other, thereby conserving time.

High PGD and mortality are also associated with increased risks of inguinal infection, venous thrombosis, and lower limb ischemia (32). Our study identified catheter-related infections despite administering prophylactic antibiotics to prevent bloodstream infections. Immunosuppressive drugs used by transplant patients increase their infection susceptibility. While definitive literature on the impact of central vs. peripheral catheterization on infection rates is scarce, central catheterization offers benefits such as high-flow unidirectional blood flow, which may reduce differential hypoxemia and inguinal infection risks (32). Matthieu et al. reported that the incidence of peripheral VA-ECMO-related infections and bleeding reached 16%, with lower limb ischemia occurring in 12% of cases (32). Reeb et al. suggested that peripheral VA-ECMO should not be considered as the primary ECMO strategy in instances of isolated pulmonary failure (33).The American Association for Thoracic Surgery (AATS) recommends preferring central VA-ECMO during transplantation (34).

Prolonged ECMO is linked to increased complications, longer hospitalization, higher costs, and greater mortality. cVA-ECMO offers an effective solution to address these challenges (35, 36). Our study found that cVA-ECMO was associated with shorter ECMO support, ICU stays, and tracheal tube removal compared to pVA-ECMO, potentially leading to lower mortality rates. However, 30-day and 1-year mortality rates did not differ significantly between the groups (Supplementary Table S1), which may be attributed to several factors. First, the relatively small sample size in the central group may have reduced statistical power, potentially masking differences in survival outcomes. Second, survival is influenced by multiple confounding factors, including the recipient's preoperative condition, donor lung quality, and postoperative infections. Although the central group exhibited a lower risk of primary graft dysfunction (PGD), these patients also had a higher baseline risk, such as a greater proportion undergoing bilateral lung transplantation, which may have offset some of the benefits. Third, PGD is an early postoperative complication, whereas long-term survival is more affected by chronic graft dysfunction and immunosuppressive-related complications, meaning that the advantage of central cannulation in reducing acute injury may not necessarily translate into improved long-term survival. Fourth, variability in surgical team experience may have influenced outcomes, as the distribution of teams across procedural groups was uneven (Table 2, *P* = 0.006). These findings align with previous studies. Ruszel et al. reported that among lung transplant recipients supported by VA-ECMO, 1-year survival rates were 66% for central cannulation and 50% for peripheral cannulation (37), noting that ECMO is often used not only as planned intraoperative support but also as an unplanned intervention for hemodynamic instability, impaired gas exchange, or right ventricular failure, all of which can impact prognosis (37). Additionally, a large meta-analysis by Biancari et al. indicated higher mortality associated with central ECMO following cardiac surgery and lung transplantation (38). In patients with postcardiotomy cardiogenic shock, Djordjevic et al. found that 30-day survival rates were comparable between central and peripheral ECMO groups (70% vs. 69%), further suggesting no definitive survival advantage based on ECMO configuration alone (39). Factors including excessive bleeding necessitating re-exploration and the administration of a large volume of blood transfusions (38), as well as cardiogenic shock, are more strongly associated with the use of VA-ECMO rather than the specific cannulation strategy employed for ECMO (39). Consequently, further research with larger sample size is needed to explore whether central cannulation provides additional long-term survival benefits compared to peripheral cannulation.

We attribute the lower PGD incidence in the central group primarily to the use of cVA-ECMO during critical periods of LTx. This approach enhances oxygenation and mitigates lung reperfusion issues before ECMO weaning. Central cannulation is particularly beneficial in the operating room for managing pulmonary hypertension and addressing surgical emergencies, such as cardiac arrest, because it can provide immediate and effective support. In contrast, the ICU typically employs a more conservative approach, favoring peripheral ECMO for stability and recovery. Matthieu supports this, showing higher immediate weaning rates in the central group (76%) compared to the peripheral group (35%), with central cannulation maintaining a low weaning rate at 6% (32).

This study has several limitations. First, we relied on the lowest oxygenation index within 72 h after surgery, a static measure that may not reflect dynamic changes in the patient's condition. Shah et al. suggest monitoring PGD fluctuations over time, identifying three phenotypes with different mortality risks (40, 41). Second, the incidence of PGD3 in the central group was higher than the 3.3% rate reported elsewhere, potentially due to the small sample size and mismatched control groups. Thirdly, as a retrospective study, we did not include a control group without ECMO. Although the number of non-ECMO cases was limited, this absence may have influenced the evaluation of ECMO's efficacy. Future prospective studies should consider incorporating a more diverse patient population to comprehensively assess the indications and prognostic outcomes associated with different support strategies. Fourth, although real-time TEE monitoring was performed intraoperatively, the data were not recorded due to limitations in the data collection system. Consequently, this important factor was not fully preserved for further analysis. In future research, we will ensure the comprehensive collection and documentation of echocardiographic data to facilitate a more direct and thorough assessment of cardiac function. Additionally, we recognize the complexity of survival outcomes and the necessity of accounting for multifactorial influences. Our study focuses on 1-year survival and does not address long-term outcomes. Moreover, as a retrospective study, certain data, including echocardiography-derived left ventricular function and end-diastolic dimensions, were not systematically collected. Hence, future randomized controlled trials are needed to validate these findings and address these limitations.

In summary, central cannulation reduces postoperative complications and improves outcomes for LTx recipients. Thus, central cannulation could enhance perioperative care, potentially leading to better recovery and long-term success. However, further prospective studies are needed to confirm these benefits and refine ECMO management protocols, ensuring that LTx patients receive optimal and safe perioperative care.

## Data Availability

The raw data supporting the conclusions of this article will be made available by the authors, without undue reservation.
