# Supplementary material for: The comparative impact of central vs. peripheral VA-ECMO cannulation on postoperative graft dysfunction in lung transplantation: a retrospective analysis
Source: Front Cardiovasc Med. 2025 Feb 25;12:1512742. doi: 10.3389/fcvm.2025.1512742 (PMC11893591; doi:10.3389/fcvm.2025.1512742)
Supplement: Supplementary file 1 [file Table1.pdf]

Supplementary Table 1. Thirty-day and 1-year survival rate after adjustment

Categories	Model 1			Model 2			Model 3		
PGD	*P*−value	OR/HR	95% CI	*P*−value	OR/HR	95% CI	*P*−value	OR/HR	95% CI
**thirty-day survival rate**									
No	Reference			Reference			Reference		
Yes	0.796	0.887	0.357-2.201	0.839	0.903	0.340-2.400	0.328	0.525	0.291-1.889
**one-year survival rate**									
No	Reference			Reference			Reference		
Yes	0.517	0.773	0.355-1.684	0.414	0.708	0.309-1.622	0.674	0.343	0.110-1.627

Model 1: Adjusted for V-A ECMO type; Model 2: Adjusted for V-A ECMO type, Sex, Age, BMI, Bilateral LTx; Model 3: Adjusted for V-A ECMO type, Surgeon Group

PGD：Primary graft dysfunction
